# Skin toxicity from external beam radiation therapy in breast cancer patients: protective effects of Resveratrol, Lycopene, Vitamin C and anthocianin (Ixor^®^)

**DOI:** 10.1186/1748-717X-7-12

**Published:** 2012-01-30

**Authors:** Rossella Di Franco, MariaGrazia Calvanese, Paola Murino, Roberto Manzo, Cesare Guida, Davide Di Gennaro, Caterina Anania, Vincenzo Ravo

**Affiliations:** 1 Dipartimento Diagnostica per Immagini e Radioterapia Seconda Università di Napoli-P.zza Luigi Miraglia-(80138) Napoli-Italy; 2 Unità Operativa Complessa Radioterapia, Presidio Ospedaliero Ascalesi ASLNA1Centro, Via Egiziaca a Forcella 31, Napoli, (80130), Italy; 3 Unità Operativa Complessa Radioterapia, AORN Moscati, Contrada Amoretta, Avellino, (83100), Italy; 4 Unità Operativa Complessa Radioterapia, Centro Aktis, Via Lazio 32, Marano (NA), (80016), Italy; 5 Unità Operativa Complessa Radioterapia, Azienda Ospedaliera Universitaria S. Giovanni di Dio e Ruggi D'Aragona, Via S.Leonardo, Salerno, (84100), Italy

**Keywords:** Radiotherapy, Breast, Toxicity, Skin

## Abstract

**Introduction:**

This is an observational study and the aim is to evaluate the effect of dietary supplements based on Resveratrol, Lycopene, Vitamin C and Anthocyanins (Ixor^®^) in reducing skin toxicity due to external beam radiotherapy in patients affected by breast cancer.

**Materials and methods:**

71 patients were enrolled and they were divided in two different groups: a control group (CG) of 41 patients treated with prophylactic topical therapy based on hyaluronic acid and topical steroid therapy in case of occurrence of radiodermatitis, and a Ixor-Group (IG) of 30 patients treated also with an oral therapy based on Resveratrol, Lycopene, Vitamin C and Anthocyanin (Ixor^®^) at a dose of 2 tablets/day, starting from 10 days before the radiation treatment until 10 days after the end of treatment. Skin toxicity has been related to PTV, to breast volume that received a radiation dose equal or lower than 107%, included between 107% and 110%, or greater than 110% of the prescribed dose. Moreover it's been studied the relationship between skin toxicity and the chemotherapy schedule used before treatment. We calculated in both groups the percentage of patients who had a skin toxicity of grade 2 or 3 (according to RTOG scale). Absolute risk reduction (ARR), relative risk (RR) and odds ratio (OR) have been calculated for each relationship.

**Results:**

Control Group (CG) patients with a PTV > 500 ml presented skin toxicity G2 + G3 in 30% of cases, versus 25% of Ixor-Group (IG) [OR 0.77]. In patients with a PTV < 500 ml G2 + G3 toxicity was 0% in the IG compared to 18% in CG (OR 0.23). When Dmax was less than or equal to 107% of the prescribed dose skin toxicity was G2 + G3 in 12.5% in CG, versus 0% in IG (OR 0.73), instead when Dmax was included between 107 and 110% of the prescribed dose, G2 + G3 skin toxicity was 35% in CG and 21% in IG (OR 0.50). In patients undergoing chemotherapy with anthracyclines and taxanes, G2 + G3 toxicity was 27% in CG, against 20% in IG (OR 0.68).

**Conclusions:**

The protective effect of Resveratrol, Lycopene, Vitamin C and Anthocyanin (Ixor^®^) is more detected in patients with PTV < 500 ml, when Dmax reaches values lower or equal to 107%, but not exceeding 110% of the prescribed dose, and in patients undergoing adjuvant chemotherapy with anthracyclines and taxanes.

## Introduction

The post-operative RT has become an integral part of the complex treatment of breast cancer. According to international standard the Radiotherapy treatment for breast cancer after conservative surgery is represented by the whole breast irradiation at the dose of 50 Gy (2 Gy/fraction), five days a week (25 fractions), subsequently an additional dose of 10 Gy must be delivered to the tumor bed (2 Gy/fraction) achieving the whole dose of 60 Gy [1,2]. In the last few years advanced radiation techniques have been developed, obtaining a better dose homogeneity within the irradiated area and a relatively low risk of local toxicity [3]. It is thought that breast IMRT would reduce by 15-20% moist desquamation in the irradiated skin, providing a greater uniformity of dose and removing hot spots [4,5], but IMRT until now is not the standard treatment for localized breast cancer.

The incidence of skin toxicity induced by radiation treatment, an important clinical problem that affects most of the patients with breast cancer undergoing adjuvant radiotherapy is related to the radiation technique, dose homogeneity, to PTV receiving a dose greater than 100% of the prescribed dose, and to prophylactic use of topical therapy. In the last years there was a growing interest in the natural substances that can have a preventive or curative role against radiation-induced dermatitis [6,7]. The use of dietary supplements such as Resveratrol, Lycopene, Vitamin C and Anthocyanins (Ixor^®^) sets in this perspective. Resveratrol (3,5,41-triidrossistilbene) is a non-flavonoid phenol of grape skin of red grapes, it has an important antioxidant effect and a dual device. It works both as a chelating agent and as a radical scavenger and in addition it takes part in inflammation by inhibiting the production of IL-8 by LPS-induced MAPK phosphorylation and a block of NF-kB activation [8,9]. The anti-inflammatory activity is also related to a relaxing effect on vessels that cause an improvement in skin microcirculation, thus finding a valuable use in irritant dermatitis [10-12]. Similarly, the Lycopene more than other carotenoids, showed a great antioxidant and anti-free radical effect, while vitamin C and a Anthocyanin have a protective effect on skin cells [13,14]. The aim of our study was to evaluate the effect of an association between topical therapies based on hyaluronic acid and dietary supplements based on Resveratrol, Lycopene, Vitamin C and Anthocyanins (Ixor^®^) in preventing acute skin toxicity in patients undergoing radiation therapy after conservative surgery for breast cancer.

## Materials and methods

Between October 2010 and June 2011, we enrolled 71 female patients with a diagnosis of breast cancer. Those patients were aged between 30 and 80 years old and they were treated, in Radiation Oncology Departments of the Ascalesi Hospital in Naples, Moscati Hospital in Avellino, Aktis Center in Marano (NA), S. Giovanni di Dio e Ruggi D'Aragona Hospital in Salerno. Ours was a retrospective observational study designed to assess the protective effect of Resveratrol, Lycopene, Vitamin C and Anthocyanins (Ixor^®^) in the skin toxicity of external beam radiotherapy directed to the breast. All enrolled patients underwent conservative surgery (quadrantectomy), surgical margins were disease-free, there wasn't indication for regional nodal radiation therapy (≤ 3 positive nodes); so these patients were candidates for adjuvant radiation treatment with 6 MV photons, with a dose of 50 Gy (2 Gy/fraction) directed to whole breast, and a subsequent additional dose of 10 Gy (2 Gy/fraction) directed to original tumor site. The patients were divided into two different groups (Table 1).

**Table 1:** Characteristics of patients enrolled in the two treatment groups.

	CG	IG
Patients (No)	41	30
**Age**		
Mean	59.71	52.10
SD (y/o)	9.92	11.26
Median	59	53
Range	(39-80)	(32-75)
**Surgery**		
Quadrantectomy	41 (100%)	30 (100%)
LAD	25 (61%)	18 (60%)
S.Linf	16 (39%)	12 (40%)
**Hystologic type**		
Ductal	35 (85%)	22 (73%)
Lobular	2 (5%)	1 (4%)
Others	4 (10%)	7 (23%)
**Chemiotherapy**		
Yes	19 (46.4%)	15 (50%)
No	22 (53.6%)	15 (50%)
**Hormone therapy**		
Yes	31 (75.6%)	21 (70%)
No	10 (24.4%)	9 (30%)
**Breast volume**		
> 500 cc	71 (75.5%)	46 (71%)
≤ 500 cc	23 (24.5%)	19 (29%)
**Breast maximum dose**		
≤ 107%	8 (19.5%)	4 (13.3%)
> 107% < 110%	26 (63.5%)	19 (63.4%)
≥ 110%	7 (17%)	7 (23.3%)

**CG**: control group **IG**: Group Ixor^®^

In the first one, the control group (CG), we enrolled 41 patients to whom was prescribed a topical prophylactic treatment based on hyaluronic acid, and a topical steroid therapy was added in case of occurrence of radiodermatitis. This treatment lasted for the entire therapy and then for the 4-6 weeks after radiation therapy. In the second group, Ixor-Group (IG), were enrolled 30 patients to whom, in addition to topic prophylactic treatment, an oral therapy based on Resveratrol, Lycopene, Vitamin C and Anthocyanins (Ixor^®^) was prescribed at a dose of 2 tablets/day. This therapy started 10 days before the radiation treatment and ended 10 days after the treatment. Inclusion criteria to be submitted to this oral therapy were: absence of breast implants and no allergy to Vitamin C. All patients were submitted to a Simulation CT-Scan and then a tridimensional care plane was set with Pinnacle^**® **^system. Target Volumes were delineated according to the International Commission of Radiation Units criteria [15,16].

The Clinical Target Volume (CTV) was defined with the entire breast tissue palpable starting from 5 mm below the skin, the Planning Target Volume (PTV) was obtained with an expansion of the CTV margin of 10 mm, except that towards the skin. The beam arrangement of treatment plan was two opposed tangential fields using beams of photons 6 mV. For each patient we calculated the Dose-Volume Histograms (DVHs) to the target and organs at risk. Each patient in either group was subjected to weekly visits during which we recorded the acute dermal toxicity according to the criteria of the Radiation Therapy Oncology Group/European Organization for Research and Treatment Cancer (RTOG/EORTC) [17]. Subsequently, to evaluate the effect of inhomogeneities on dose toxicity, we related, in both groups, skin toxicity to PTV, but also to dosimetric factors, such as the breast volume receiving a dose equal or less than 107% of the prescribed dose, the one that received a dose greater than 107% and lower than 110% of the prescribed dose, and patients receiving a dose greater than 110% of the prescribed dose. All the doses > 107% are to be considered as single points in which there is a maximum dose. Finally, it was evaluated, in two groups, the skin toxicity in relation to the chemotherapy schedule to which patients were submitted. In all cases, acute dermatitis related to radiation therapy was calculated from the percentage of patients presenting acute dermal toxicity of grade 2 or 3 (G2 + G3 RTOG). For each report it has been calculated the absolute risk reduction (ARR), relative risk (RR) and odds ratio (OR).

## Results

Starting from data collected, the first relationship we made regarded acute dermal toxicity and PTV (Table 2).

**Table 2:** Number of patients with mild skin toxicity (G0+G1) and high grade (G2+G3) observed in the two groups of patients distinguished according to the PTV

PTV > 500 ml			PTV < 500 ml	
	CG	IG	CG	IG
**Tox (RTOG)**				
**Grade (0+1)**	21 pz (70%)	15 pz (75%)	9 pz (82%)	10 pz (100%)
**Grade (2+3)**	9 pz(30%)	5 pz (25%)	2 pz (18%)	0 pz (0%)

When PTV was > 500 ml, Control Group (CG) showed a high toxicity (G2 + G3) in 30% of cases if compared with 25% of cases of Ixor^®^-Group (IG), with ARR of 5%, RR of 0.83 and OR equal to 0.77. When PTV was < 500 ml, the CG presented a skin toxicity (G2 + G3) in 18% of cases, versus 0% of IG, with ARR of 18%, RR of 0.28 and OR equal to 0.23.

Then we related skin toxicity to dosimetric values of each treatment plan (Table 3).

**Table 3:** Number of patients with mild skin toxicity (G0+G1) and high grade (G2+G3) observed in two distinct groups of patients based on the maximum dose absorbed

Maximum dose < 107%			Maximum dose > 107% < 110%		Maximum dose > 110%	
	CG	IG	CG	IG	CG	IG
Tox (RTOG)						
Grade (0+1)	7 pz (87.5%)	4 pz (100%)	17 pz (65%)	15 pz (79%)	6 pz (86%)	6 pz (86%)
Grade (2+3)	1 pz (12.5%)	0 pz (0%)	9 pz (35%)	4 pz (21%)	1 pz (14%)	1 pz (14%)

In patients receiving a maximum dose less or equal to 107% of the prescribed, CG presented a toxicity G2+G3 in 12.5% of cases, compared with 0% of IG with ARR 12.5%, RR of 0.77 and OR of 0.73.

In patients who received a maximum dose higher than 107% and lower than 110% of the prescribed, CG presented a high skin toxicity (G2+G3) in 35% of cases versus 21% of the IG, with ARR of 14%, RR of 0.60 and OR of 0.50.

In patients receiving a maximum dose higher than 110% of the prescribed dose, the CG and the IG showed the same percentage of toxicity G2+G3 with RR and OR of 1.

We also evaluated skin toxicity dividing patients in two different groups distinguishing between patients who underwent radiation therapy alone as adjuvant treatment, and patients who were submitted to an adjuvant treatment based on chemotherapy and radiotherapy. Particularly it was evaluated the protective effect of Resveratrol, Lycopene, Vitamin C and Anthocyanins (Ixor^®^) in patients undergoing chemotherapy with Anthracyclines and Taxanes, that is a treatment schedule marked by an higher skin toxicity (Table 4).

**Table 4:** Number of patients with mild skin toxicity (G0+G1) and high grade (G2+G3) observed in patients undergoing CT and CT in patients undergoing second scheme anthracyclines and taxanes

NO CT			CT (Anthracyclines + Taxanes)	
	CG	IG	CG	IG
Tox (RTOG)				
Grade (0+1)	15 pz (68%)	14 pz (93%)	8 pz (73%)	4 pz (80%)
Grade (2+3)	7 pz (32%)	1 pz (7%)	3 pz (27%)	1 pz (20%)

Results suggested a protective effect of Resveratrol, Lycopene, Vitamin C and Anthocyanins (Ixor^®^) in both groups with ARR 25%, RR of 0.22 and OR of 0.17 in patients who were not submitted to chemotherapy, and with ARR 7%, RR of 0.74 and OR of 0.68 in patients who underwent chemotherapy with Antracyclines and Taxanes.

**Figure 1:**
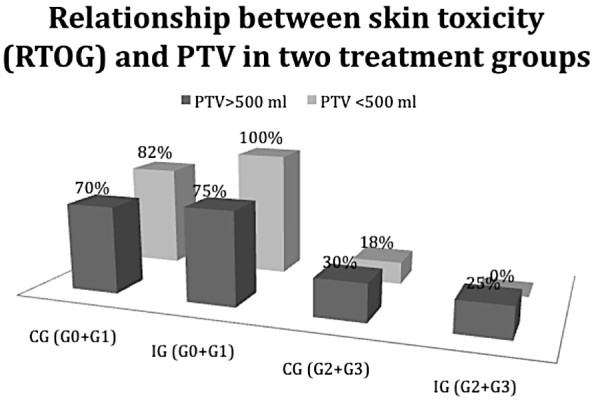
**Relationship between the PTV (> 500 ml or < 500 ml) and the percentage of patients with mild toxicity (G0+G1) or severe toxicity (G2+G3) in the two treatment groups**.

**Figure 2:**
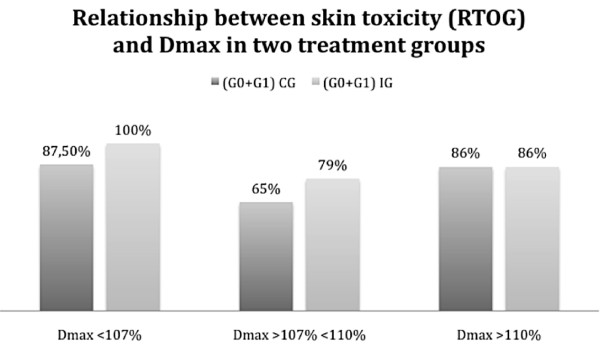
**Relationship between the Dmax (< 107%; > 107% < 110%; > 110%) and the percentage of patients with mild toxicity (G0+G1) in the two treatment groups**.

The relationships described are described in Figure 1 and 2.

In Figure 1 it is expressed the relationship between skin toxicity and PTV reported in two treatment groups. As shown, when PTV was > 500 ml we had an higher reduction of G2+G3 skin toxicity percentage in IG if compared to CG, but this reduction is outstanding in patients with a PTV < 500 ml. In this case, we observed 0% of high skin toxicity in IG versus 18% of cases recorded in the CG.

In Figure 2 it is expressed the relationship between mild skin toxicity (G0+G1) and Dmax reported in two treatment groups. As shown there is a significant prevalence of mild toxicity in the IG when Dmax reaches values lower or equal to 107% of the prescribed dose and when values of 110% of the dose are not exceeded. However when Dmax exceeds 110% of the dose there is no difference in toxicity, between the two treatment groups.

**Figure 3:**
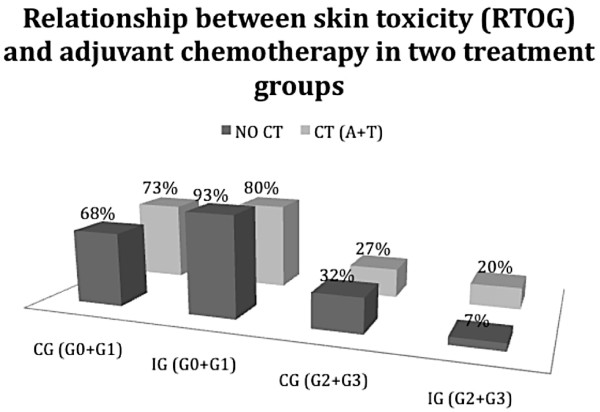
**Relationship between the type of adjuvant chemotherapy (NO CT and A+T) and the percentage of patients with skin toxicity in the two treatment groups**.

The graphic in Figure 3 shows the protective effect of Resveratrol, Lycopene, Vitamin C and Anthocyanins (Ixor^®^) in patients without CT adjuvant treatments, but mainly in patients undergoing chemotherapy with Anthracyclines and Taxanes, which is a recognized scheme with a greater skin toxicity.

## Discussion

Today women ask us not only to be cured but also the preservation of their body; this is one of our most important endpoints in modern radiotherapy treatments. The radiotherapy-induced skin toxicity remains a major clinical problem that affects many patients with breast cancer undergoing adjuvant radiotherapy [18]. Anyway there is a growing emphasis on prevention and treatment of side effects induced by radiation therapy; this attention has derived many studies dealing about the treatments and substances that can protect healthy organs from radiation therapy side effects.

Surely the breast volume has a great impact on the incidence and the level of skin toxicity, but also the breast volume receiving dose-points equal or greater than 107% of the one prescribed has an impact in the arising of side effects. That's because many studies aimed to find appropriate prevention methods for damages are born. These methods seemed to be helpful in 25% of patients with breast cancer who presented a high desquamation and ulceration of skin due to Radiation therapy. Particularly, Chen et al. in their study, evaluated the effects of cutaneous prophylactic treatment on dosimetry and toxicity due to radiation therapy, demonstrating a correlation between skin toxicity and a dose equal to or greater than 107% or equal to 110% of the prescribed dose [18]. In fact they showed that the presence of a large volume receiving more than 53.9 Gy, or more than 55.4 Gy, was a predisposing factor to the onset of radiotherapy-induced skin toxicity. The same study suggests that prophylactic treatment with steroids can help the skin to prevent dermatitis induced by higher radiation dose, demonstrating a protective effect of treatment with steroid creams in those cases where there was a lack of homogeneity of radiation dose [3,19]. It's known by a long time the preventing role of a diet rich in antioxidants with regard to pathological conditions such as cancer, atherosclerosis, stroke, neurodegenerative diseases, diabetes [20,21].

It was also highlighted the radioprotective effect of some components of wine. Morganti et al. evaluated the capability of red wine in preventing the effects of acute toxicity in patients undergoing radiation therapy after conservative surgery for breast cancer. In particular, they evaluated the protective effects of wine components in the context of different regimes of radiotherapy and adjuvant treatments such as chemotherapy or hormone therapy, showing a moderate protective effect of wine against the degree of acute toxicity [22].

Our study fits into this field of interest because it evaluates the radioprotective effect of natural substances such as Resveratrol (RV) present in grape skin of red grapes, the Lycopene, a carotenoid that has a high antioxidant capacity and anti free-radical action, and Vitamin C and Anthocyanins, contained in the red orange.

Other studies evaluated the effects of RV. Some has demonstrated its effect on the production of IL-8 in human monocytic line demonstrating that RV inhibits its production arresting MAPK phosphorylation and activating NF-kB, there by modulating THP-1 [23]. In a review of Reagan et al. it was reported the usefulness of the RV in preventing damage caused by UVB radiation and its ability to improve the radiosensitivity of tumor cell lines [24]. Other studies have shown its anti-cancer effect, it seems that the RV inhibits tumorogenesis through the induction of apoptosis and the activation of genes FAS, p-53 and p21 [25,26].

Our study focuses his attention in the first step of the pathophysiology underlying the onset of dermatitis due to radiation therapy, namely the excessive production of free radicals due to radiolysis of water, responsible for damage to the cells of the basal layer of the epidermis and endothelial cells. Substances with anti-inflammatory and antioxidant effects have been considered good candidates for protection against radiodermatitis, so we evaluated the potential protective role of Resveratrol, the Lycopene, Vitamin C and Anthocyanins (Ixor^®^), and we found encouraging results in breasts with a volume lower than 500 ml and in those who receive a radiation dose between 107% and 110% of the prescribed dose. The probability to record some "hot spots" in a treatment planning, with points in the PTV receiving doses > 107%, is high particularly in patients with breasts > 500 cc. Probably the toxicity linked to these, sometimes inevitable, dose uncertainties can't be avoided by any concomitant medical treatment. In fact we found no difference in the side effects of patients treated with this planning-dose characteristics. But in patients with a dose distribution that respects the conventional dose-constraints an advantage was found in the IG group. Moreover the use of chemotherapy, alone or in combination, represents a major therapeutic strategy for malignant tumors, but it is inevitably linked to some adverse events that include dermal toxicity, especially with regard to the pattern with Anthracyclines and Taxanes [27]. Also in these patients the IG had a better local result if compared to the CG. In our study we focused our interest also on this aspect, demonstrating a protective value of Resveratrol, Lycopene, Vitamin C and Anthocyanins (Ixor^®^) in patients undergoing chemotherapy with Anthracyclines and Taxanes. Obviously, this is a preliminary study, and has some limitations, first of all, the limited number of patients.

Our purpose is to evaluate the protective effect of Resveratrol, the Lycopene, Vitamin C and Anthocyanins (Ixor^®^) on a larger number of patients, and to evaluate its action in early follow-up in order to study its action not only in the prevention of in high-grade dermatitis but also in returned earlier curing of the mammary skin after radiation therapy.

## Competing interests

The authors declare that they have no competing interests.

## Authors' contributions

R.DF., MG.C., performed the literature search, extracted relevant articles and drafted the manuscript, P.M., R.M. and V.R. contributed equally to this work participating in the design and coordination of the study, C.G., D.DG. and C.A. participated in the design of the study. All authors read and approved the final manuscript.
